# Donor-size-normalized tidal volume and early peak airway pressure-derived pressure burden after lung transplantation: an exploratory single-center cohort study

**DOI:** 10.3389/fmed.2026.1873272

**Published:** 2026-07-23

**Authors:** Tao Wang, Yang Zhou, Xue Li, Jing Hu, Ding Huang, Weidong Wang, Rui Li, Junrong Ding

**Affiliations:** 1 Department of Thoracic Surgery, Shanghai Pulmonary Hospital, Tongji University School of Medicine, Shanghai, China; 2 School of Medicine, Tongji University, Shanghai, China; 3 Department of Nursing, Shanghai Pulmonary Hospital, Tongji University School of Medicine, Shanghai, China

**Keywords:** donor-predicted body weight, dynamic compliance, lung transplantation, mechanical ventilation, peak airway pressure, primary graft dysfunction, tidal volume, ventilator-induced lung injury

## Abstract

**Introduction:**

After lung transplantation, the ventilated parenchyma is the donor lung rather than the recipient's native lung. Normalizing tidal volume to donor-predicted body weight (dPBW) indexes delivered ventilation to estimated graft size.

**Methods:**

We conducted a retrospective single-center cohort study to evaluate whether dPBW-normalized tidal volume at intensive care unit admission after transplantation (T0) was associated with time-matched peak pressure difference under clinician-selected ventilation. Peak pressure difference was defined as peak airway pressure minus positive end-expiratory pressure and served as a peak airway pressure-derived pressure-burden metric rather than standard driving pressure.

**Results:**

Among 85 adult lung transplant recipients in the primary complete-case analysis, higher T0 dPBW-normalized tidal volume was associated with lower T0 peak pressure difference after enhanced adjustment (β = −0.531, 95% CI −1.031 to −0.032; *P* = 0.0376). Secondary analyses of dynamic compliance showed an association with higher time-matched dynamic compliance. In contrast, dPBW-normalized tidal volume did not show significant associations with severe primary graft dysfunction, liberation from invasive mechanical ventilation within 28 days, or 30-day mortality after enhanced adjustment.

**Discussion:**

These exploratory findings describe associations between dPBW-normalized tidal volume and early peak airway pressure-derived burden under clinician-selected ventilation, but they do not establish a physiologic effect or support changes in ventilatory management. Prospective validation with protocolized ventilation and plateau-pressure measurements is required.

## Introduction

Lung transplantation is an established treatment for selected patients with end-stage lung disease (1, 2). Recent studies have further highlighted the growing clinical relevance of lung transplantation (3, 4). However, early graft injury remains a major perioperative challenge, and primary graft dysfunction (PGD) is a standardized early graft-injury phenotype after transplantation (5, 6). Cohort and prediction studies have identified donor, recipient, and perioperative risk factors for PGD (7–9). During the first h after implantation, the donor lung is exposed to ischemia–reperfusion injury, permeability edema, atelectasis, altered chest wall mechanics, and changes in pulmonary vascular tone (10–12). Inflammatory, complement, and endothelial injury pathways may further shape early graft behavior (13–15). Mechanical ventilation is required to support gas exchange and graft recruitment during this period. At the same time, excessive volume or pressure loading may increase mechanical stress and contribute to ventilator-induced lung injury (16–18). Modern respiratory support guidance and mechanical power concepts reinforce the importance of limiting injurious ventilation exposure (19, 20). An accurate description of early postoperative ventilation exposure is important in lung transplantation.

Tidal volume is central to protective ventilation and is usually indexed to predicted body weight (17–19). In most critically ill adults, this denominator is derived from the patient being ventilated. Lung transplantation creates a distinct physiologic setting. The ventilated parenchyma is the donor lung, not the recipient's native lung. Ventilation practice after lung transplantation is heterogeneous and commonly individualized to graft size, gas exchange, airway pressures, and perioperative support (21, 22). When donor and recipient body sizes differ, the same absolute tidal volume may represent different mechanical exposure depending on whether it is normalized to recipient-predicted body weight (rPBW) or donor-predicted body weight (dPBW) (23, 24). Size-matching studies also suggest that donor–recipient size relationships may influence ventilation exposure, PGD, and post-transplant outcomes (25–28). These considerations support examining tidal volume indexed to dPBW as a marker of ventilation exposure relative to estimated graft size after transplantation.

Previous studies of donor-normalized ventilation and donor–recipient size matching after lung transplantation have mainly focused on downstream clinical outcomes, including PGD, ventilator-free days, and survival (28–30). These outcomes are clinically important, but they are influenced by many perioperative and postoperative factors beyond early ventilation exposure (31–33). Prolonged ventilation after lung transplantation is also shaped by multiple recipient, donor, operative, and postoperative factors (34, 35). Early bedside respiratory mechanics may provide a more proximal physiologic readout of the relationship between donor-size-normalized ventilation exposure and early graft behavior. Dynamic compliance measured immediately after intensive care unit admission reflects respiratory system behavior under ongoing postoperative ventilation. It incorporates graft mechanics, recruitment status, airway pressures, edema, and chest wall effects. However, dynamic compliance is also influenced by ventilator settings and includes tidal volume in its calculation. This creates an important risk of mathematical coupling when the exposure is based on tidal volume. A pressure-related endpoint may provide useful additional context, although it cannot replace direct plateau pressure or standard driving pressure measurements (36–38).

We conducted a retrospective single-center cohort study to evaluate whether T0 dPBW-normalized tidal volume under clinician-selected ventilation was associated with T0 peak pressure difference after lung transplantation. We also examined dynamic compliance, early gas-exchange variables, major postoperative clinical outcomes, ECMO-related heterogeneity, and donor–recipient size matching.

## Materials and methods

### Study design, setting, and ethics

This retrospective single-center cohort study was conducted at Shanghai Pulmonary Hospital, a national tertiary referral center for lung transplantation in Shanghai, China. Consecutive adult patients who underwent lung transplantation between 3 June 2022 and 4 June 2024 were screened for eligibility. Clinical, donor, perioperative, and early postoperative ventilation data were extracted from an institutional database and supplemented by review of the electronic medical record.

The study was approved by the Ethics Committee of Shanghai Pulmonary Hospital (approval No. Q24-713; protocol No. 01). It was conducted in accordance with institutional requirements, the Declaration of Helsinki, and the Declaration of Istanbul (39, 40). Because this retrospective study used routinely collected clinical data, the requirement for written informed consent was waived by the ethics committee. Donor organs were procured in accordance with applicable laws and institutional policies. No organs or tissues were obtained through illegal commercial transactions or from executed prisoners.

### Study population

Patients were eligible if they were 18 years of age or older and underwent single or bilateral lung transplantation during the study period. Recipients who underwent retransplantation were excluded. Patients were included in the primary analytic cohort if T0 donor-predicted body weight (dPBW)-normalized tidal volume, T0 peak pressure difference, and all covariates required for the primary regression models were available. No *a priori* sample-size calculation was performed; the cohort size was determined by consecutive eligibility during the predefined study period.

### Perioperative ventilation management

Postoperative mechanical ventilation was managed according to the institutional lung-transplant ICU protocol. After ICU admission, volume-controlled or pressure-controlled ventilation was selected by the attending intensivist and the transplant anesthesiology team. Ventilator settings generally followed protective ventilation principles and were individualized according to estimated graft size, gas exchange, airway pressure, and hemodynamic status (21, 22). Positive end-expiratory pressure (PEEP), fraction of inspired oxygen (FiO_2_), respiratory rate, and inspiratory pressure were adjusted according to oxygenation, carbon dioxide clearance, airway pressures, chest radiographic findings, and extracorporeal membrane oxygenation (ECMO) support. In recipients receiving postoperative ECMO, ventilator settings were individualized to reduce mechanical stress while maintaining gas exchange and graft recruitment (41). Analgesia, sedation, fluid management, and vasoactive support were managed by the ICU team in accordance with institutional practice.

The ventilation mode reflected usual clinical care and was not assigned by protocol. At T0, ventilation mode was abstracted from the ICU ventilator record and categorized as controlled mechanical ventilation (CMV), pressure-controlled ventilation (PCV), pressure-synchronized intermittent mandatory ventilation (PSIMV), or synchronized intermittent mandatory ventilation (SIMV). T0 positive end-expiratory pressure (PEEP) was abstracted from the same time point and summarized descriptively.

### Exposure assessment

The primary exposure was tidal volume normalized to dPBW at intensive care unit (ICU) admission after transplantation (T0), expressed as mL/kg dPBW. This metric was used as a donor-size-normalized ventilation exposure measure. It describes delivered tidal volume relative to estimated graft size. Donor-predicted body weight was calculated using sex-specific predicted body weight formulas:


$$\begin{array}{l} dPBW=\{\begin{array}{c} 50+0.91\times \left(\text{donor height }\left[\text{cm}\right]-152.4\right),\text{ male},\\ 45.5+0.91\times \left(\text{donor height }\left[\text{cm}\right]-152.4\right),\text{ female}. \end{array} \end{array}$$

Recipient-predicted body weight (rPBW) was calculated analogously using recipient sex and height:


$$\begin{array}{l} rPBW=\{\begin{array}{c} 50+0.91\times \left(\text{recipient height }\left[\text{cm}\right]-152.4\right),\text{ male},\\ 45.5+0.91\times \left(\text{recipient height }\left[\text{cm}\right]-152.4\right),\text{ female}. \end{array} \end{array}$$

These formulas are consistent with predicted body weight-based protective-ventilation conventions (17, 18). Donor–recipient size matching was quantified as the dPBW/rPBW ratio. For categorical size-mismatch summaries, this ratio was categorized as marked donor undersizing (dPBW/rPBW < 0.8), approximate size matching (dPBW/rPBW 0.8–1.2), or marked donor oversizing (dPBW/rPBW > 1.2) (24–27).

Postoperative tidal volume was assessed at ICU admission after transplantation (T0) and 24 h after transplantation (T24). To maintain temporal alignment between exposure and outcome, the primary analysis used T0 dPBW-normalized tidal volume as the exposure for T0 peak pressure difference. Secondary analyses evaluated T0 dPBW-normalized tidal volume, the dPBW/rPBW ratio, and mean dPBW-normalized tidal volume during the first 24 postoperative h in relation to T0 dynamic compliance. Additional time-matched analyses used T0 and T24 dPBW-normalized tidal volumes for corresponding respiratory-mechanics and oxygenation endpoints.

### Outcomes

The primary outcome was T0 peak pressure difference, calculated as T0 peak airway pressure minus T0 PEEP immediately after ICU admission. This endpoint represents an early peak airway pressure-derived pressure-burden metric rather than standard driving pressure. Standard driving pressure, transpulmonary pressure, and mechanical power require measurements that were not systematically available in these routine records.

Secondary physiologic outcomes were T0 and T24 dynamic compliance, T0 and T24 PaO_2_/FiO_2_ ratio, T0 and T24 FiO_2_, T0 and T24 arterial oxygen tension (PaO_2_), and T0 and T24 arterial carbon dioxide tension (PaCO_2_). These outcomes characterized early respiratory mechanics, oxygenation, and ventilation efficiency during the immediate postoperative period. Dynamic compliance includes tidal volume in its calculation and may therefore be mathematically coupled to dPBW-normalized tidal volume.

Clinical secondary outcomes were severe primary graft dysfunction (PGD) at 48 and/or 72 h after transplantation, liberation from invasive mechanical ventilation within 28 days, and 30-day mortality. Severe PGD was defined according to the 2016 International Society for Heart and Lung Transplantation consensus criteria as Grade 3 PGD at 48 and/or 72 h after transplantation (5). PGD grade was assessed at T0, T24, T48, and T72 using postoperative chest radiographic findings and oxygenation status. In the absence of chest radiographic evidence of pulmonary edema, PGD was classified as Grade 0. When pulmonary edema was present, PGD grade was assigned according to the corresponding PaO_2_/FiO_2_ ratio (5, 6).

### Covariates

Covariates were selected on clinical grounds. The minimally adjusted model included recipient age, recipient sex, body mass index, and transplant type. For the primary T0 peak pressure difference analysis, the enhanced adjustment model additionally included preoperative primary diagnosis group, preoperative invasive mechanical ventilation, preoperative ECMO, postoperative ECMO within 24 h, ischemic time, intraoperative transfusion, intraoperative cardiopulmonary bypass or ECMO support, T0 FiO_2_, and donor age. T0 PEEP was omitted from the enhanced model because the outcome was defined as peak airway pressure minus PEEP. For secondary respiratory-mechanics analyses, the enhanced adjustment model additionally included the same covariates, plus T0 PEEP, when the outcome did not include PEEP in its definition. For binary clinical outcomes, the enhanced adjustment model included the minimally adjusted covariates plus preoperative primary diagnosis group, postoperative ECMO within 24 h, ischemic time, T0 FiO_2_, and donor age.

### Statistical analysis

Continuous variables were summarized as the median and interquartile range (IQR). Categorical variables were summarized as counts and percentages. Baseline characteristics were summarized overall and, where relevant, according to severe PGD status at 48–72 h. Between-group comparisons used the Mann–Whitney U-test for continuous variables and Fisher's exact test for categorical variables.

Associations between donor-size-normalized ventilation exposure and continuous physiologic outcomes were evaluated using linear regression. The primary model estimated the association between T0 dPBW-normalized tidal volume and T0 peak pressure difference. Three model layers were fitted for the primary outcome: an unadjusted model, a minimally adjusted model, and an enhanced adjusted model.

Secondary physiologic analyses used linear regression to evaluate associations between dPBW-normalized tidal volume and time-matched dynamic compliance, PaO_2_/FiO_2_ ratio, FiO_2_, PaO_2_, and PaCO_2_. For T0 dynamic compliance, exposure analyses evaluated T0 dPBW-normalized tidal volume, the dPBW/rPBW ratio, and mean 0–24 h dPBW-normalized tidal volume.

Associations between ventilation exposure metrics and fixed-window binary clinical outcomes were evaluated using logistic regression. Logistic models were fitted for severe PGD at 48–72 h, liberation from invasive mechanical ventilation within 28 days, and 30-day mortality. For these clinical endpoints, T0 dPBW-normalized tidal volume, the dPBW/rPBW ratio, and mean 0–24 h dPBW-normalized tidal volume were examined in parallel model sets.

Supplementary analyses included time-matched validation models relating T24 dPBW-normalized tidal volume to T24 dynamic compliance and models evaluating T0 and T24 PaO_2_/FiO_2_ ratio. Additional supplementary linear models evaluated gas-exchange variables, including FiO_2_, PaO_2_, and PaCO_2_ at T0 and T24.

Donor–recipient size-matching analyses evaluated whether the dPBW/rPBW ratio modified the association between T0 dPBW-normalized tidal volume and T0 dynamic compliance. Effect modification was assessed by adding a product term between T0 dPBW-normalized tidal volume and the continuous dPBW/rPBW ratio to the dynamic compliance linear regression model. The dPBW/rPBW categories (< 0.8, 0.8–1.2, and >1.2) were summarized descriptively, and stratum-specific slopes were estimated within these categories.

Restriction analyses repeated the dynamic compliance models after excluding recipients with postoperative ECMO within 24 h, excluding recipients who received intraoperative cardiopulmonary bypass or ECMO support, and excluding both perioperative extracorporeal-support groups simultaneously. Additional interaction analyses evaluated whether postoperative ECMO within 24 h modified this association by adding an interaction term between T0 dPBW-normalized tidal volume and postoperative ECMO status.

Additional stratum-specific analyses described the primary pressure-burden association within single lung and bilateral lung transplantation strata and within postoperative ECMO strata. Unadjusted interaction terms evaluated whether transplant type or postoperative ECMO status modified the association between T0 dPBW-normalized tidal volume and T0 peak pressure difference.

To evaluate potential heterogeneity related to ventilatory mode, an additional exploratory sensitivity analysis was performed by restricting the primary pressure-burden analysis to recipients recorded as receiving CMV at T0. Because this restricted stratum was small, unadjusted and minimally adjusted linear models were fitted using the same minimally adjusted covariate set as in the primary analysis, whereas enhanced adjustment was not applied.

Effect estimates from linear models are reported as regression coefficients (β) with 95% confidence intervals (CIs). Effect estimates from logistic models are reported as odds ratios (ORs) with 95% CIs. Complete-case analysis was used for all regression models (42). The number of participants included in each model is reported in the corresponding main or Supplementary Table. Missingness of key exposure, outcome, and covariate variables is summarized in Supplementary Table S1. Two-sided *P*-values < 0.05 were considered statistically significant. No adjustment was made for multiple comparisons; secondary and subgroup analyses were reported descriptively (43). All analyses were performed using R software (version 4.5.2; R Foundation for Statistical Computing, Vienna, Austria). The study is reported in accordance with the STROBE guideline for observational studies (44).

## Results

### Study population

**Figure 1:**
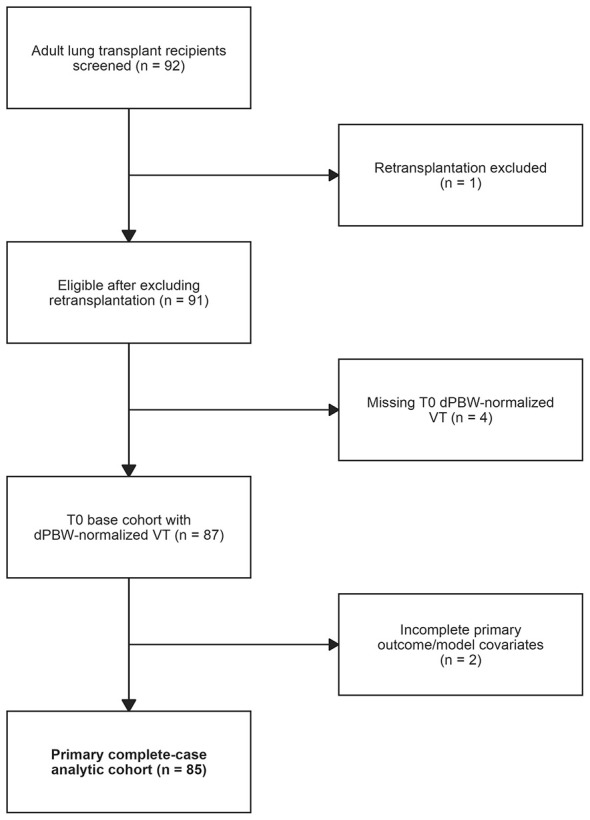
Study flow diagram. Adult lung transplant recipients were screened from 3 June 2022 to 4 June 2024. The central boxes show the sequential analysis cohorts, and the right-hand callouts show exclusions or missing-data filters at each step. After exclusion of one retransplantation case, 91 recipients were eligible, 87 had T0 dPBW-normalized tidal-volume data for the T0 base cohort, and 85 recipients comprised the primary complete-case analytic cohort. Missing data for the primary complete-case analysis included missing T0 dPBW-normalized tidal volume, T0 peak pressure difference, or covariates required for the primary regression models. dPBW, donor-predicted body weight; VT, tidal volume.

Of 92 screened adult lung transplant recipients, excluding one retransplantation case left 91 eligible recipients. Of these, 87 had T0 donor-size-normalized tidal-volume data for the T0 base cohort, and 85 entered the primary complete-case analytic cohort (Figure 1). Baseline characteristics of the T0 base cohort are summarized in Table 1. Missingness of key exposure, outcome, and covariate variables is summarized in Supplementary Table S1. The cohort included recipients who required perioperative extracorporeal support.

**Table 1:** Baseline characteristics of the T0 base cohort.

Characteristic	Overall (*n* = 87)	No severe PGD (*n* = 34)	Severe PGD (*n* = 53)
Recipient age, years	65.0 (58.0–68.0)	65.0 (59.0–68.0)	65.0 (58.0–69.0)
Body mass index, kg/m2	22.8 (20.5–24.6)	21.9 (19.5–23.9)	22.9 (21.5–24.9)
Ischemic time, min	470.0 (420.0–510.0)	475.0 (420.0–486.0)	470.0 (420.0–510.0)
Donor age, years	45.0 (35.0–51.0)	45.0 (35.0–52.0)	44.0 (35.5–49.5)
Donor BMI, kg/m2	22.9 (20.8–24.2)	23.4 (21.2–25.4)	22.5 (20.8–23.7)
dPBW/rPBW ratio	1.0 (0.9–1.2)	1.0 (0.9–1.1)	1.0 (0.9–1.2)
Recipient sex, *n* (%)			
Female	13 (14.9%)	4 (11.8%)	9 (17.0%)
Male	74 (85.1%)	30 (88.2%)	44 (83.0%)
Transplant type, *n* (%)			
Single	46 (52.9%)	19 (55.9%)	27 (50.9%)
Bilateral	41 (47.1%)	15 (44.1%)	26 (49.1%)
Preoperative diagnosis, *n* (%)			
Interstitial pneumonia	40 (46.0%)	11 (32.4%)	29 (54.7%)
COPD	28 (32.2%)	15 (44.1%)	13 (24.5%)
Pulmonary fibrosis	13 (14.9%)	4 (11.8%)	9 (17.0%)
Other	4 (4.6%)	3 (8.8%)	1 (1.9%)
Bronchiectasis	1 (1.1%)	0 (0.0%)	1 (1.9%)
Pulmonary hypertension	1 (1.1%)	1 (2.9%)	0 (0.0%)
Preoperative IMV, *n* (%)			
No	64 (73.6%)	27 (79.4%)	37 (69.8%)
Yes	23 (26.4%)	7 (20.6%)	16 (30.2%)
Preoperative ECMO, *n* (%)			
No	69 (79.3%)	28 (82.4%)	41 (77.4%)
Yes	18 (20.7%)	6 (17.6%)	12 (22.6%)
Postoperative ECMO within 24 h, *n* (%)			
No	47 (54.0%)	23 (67.6%)	24 (45.3%)
Yes	40 (46.0%)	11 (32.4%)	29 (54.7%)
Intraoperative transfusion, *n* (%)			
No	24 (27.6%)	12 (35.3%)	12 (22.6%)
Yes	63 (72.4%)	22 (64.7%)	41 (77.4%)
Intraoperative CPB or ECMO, *n* (%)			
No	43 (49.4%)	20 (58.8%)	23 (43.4%)
Yes	44 (50.6%)	14 (41.2%)	30 (56.6%)
Donor sex, *n* (%)			
Female	12 (13.8%)	7 (20.6%)	5 (9.4%)
Male	75 (86.2%)	27 (79.4%)	48 (90.6%)
Size mismatch group, *n* (%)			
Matched (0.8–1.2)	66 (75.9%)	27 (79.4%)	39 (73.6%)
Undersizing (<0.8)	7 (8.0%)	4 (11.8%)	3 (5.7%)
Oversizing (>1.2)	14 (16.1%)	3 (8.8%)	11 (20.8%)

Data are presented as median (interquartile range) or *n* (%). PGD, primary graft dysfunction; BMI, body mass index; dPBW, donor-predicted body weight; rPBW, recipient-predicted body weight; COPD, chronic obstructive pulmonary disease; IMV, invasive mechanical ventilation; ECMO, extracorporeal membrane oxygenation; CPB, cardiopulmonary bypass. Severe PGD was defined as Grade 3 PGD at 48 and/or 72 h after transplantation. Percentages may not sum to 100 because of rounding.

In the T0 base cohort, T0 ventilation mode was controlled mechanical ventilation (CMV) in 40 recipients (46.0%), pressure-controlled ventilation (PCV) in 30 (34.5%), pressure-synchronized intermittent mandatory ventilation (PSIMV) in 12 (13.8%), and synchronized intermittent mandatory ventilation (SIMV) in 5 (5.7%). T0 PEEP was available for all 87 recipients; the median T0 PEEP was 5.0 cmH_2_O (IQR, 5.0–8.0; range, 5.0–10.0).

### Primary association with the T0 peak pressure difference

**Figure 2:**
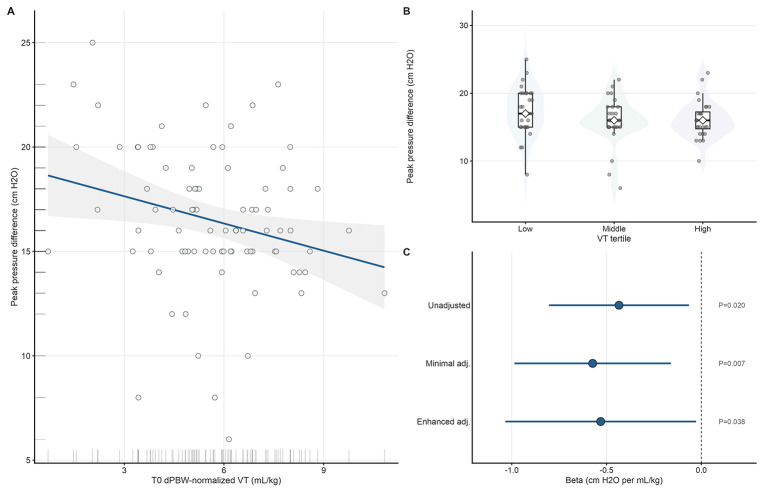
Primary association between T0 donor-size-normalized tidal volume and T0 peak airway pressure-derived pressure burden. **(A)** Continuous association between T0 dPBW-normalized tidal volume and T0 peak pressure difference in the primary complete-case analytic cohort (*n* = 85). The fitted line represents a descriptive ordinary least-squares fit with a 95% confidence band; prespecified inferential models treated T0 dPBW-normalized tidal volume as a continuous exposure. **(B)** Raincloud-style distribution of T0 peak pressure difference across tertiles of T0 dPBW-normalized tidal volume. Tertiles are shown for visualization only. **(C)** Regression coefficients for the primary pressure-burden association across unadjusted, minimally adjusted, and enhanced adjusted models. In panel C, points denote regression coefficient estimates, horizontal lines denote 95% confidence intervals, and the vertical dashed line denotes the null value of 0; rounded *P*-values are displayed in panel C; exact effect estimates, exact *P*-values, and complete-case sample sizes are provided in Table 2. T0 peak pressure difference was calculated as peak airway pressure minus positive end-expiratory pressure and represents a peak airway pressure-derived pressure-burden metric rather than standard driving pressure. dPBW, donor-predicted body weight; VT, tidal volume. The fitted line and tertile display are included for visualization.

T0 dPBW-normalized tidal volume was associated with lower T0 peak pressure difference in the unadjusted model (β = −0.434, 95% CI −0.800 to −0.069, *P* = 0.0204), minimally adjusted model (β = −0.573, 95% CI −0.982 to −0.165, *P* = 0.0066), and enhanced adjusted model (β = −0.531, 95% CI −1.031 to −0.032, *P* = 0.0376; Table 2; Figure 2).

**Table 2:** Primary association between T0 dPBW-normalized tidal volume and T0 peak pressure difference.

Model	β	95% CI	*P*-value	*n*
Unadjusted	−0.434	−0.800 to −0.069	0.0204	85
Minimally adjusted	−0.573	−0.982 to −0.165	0.0066	85
Enhanced adjusted	−0.531	−1.031 to −0.032	0.0376	85

dPBW, donor-predicted body weight; CI, confidence interval; PEEP, positive end-expiratory pressure; FiO_2_, fraction of inspired oxygen. The exposure was T0 dPBW-normalized tidal volume. β is expressed as the change in T0 peak pressure difference (cmH_2_O) per 1 mL/kg dPBW higher T0 dPBW-normalized tidal volume. T0 peak pressure difference was defined as T0 peak airway pressure minus T0 PEEP. This endpoint represents a peak airway pressure-derived pressure-burden metric rather than standard driving pressure. The enhanced adjusted model included recipient age, recipient sex, body mass index, transplant type, preoperative primary diagnosis group, preoperative invasive mechanical ventilation, preoperative extracorporeal membrane oxygenation, postoperative extracorporeal membrane oxygenation within 24 h, ischemic time, intraoperative transfusion, intraoperative cardiopulmonary bypass or extracorporeal membrane oxygenation support, T0 FiO_2_, and donor age. T0 PEEP was not included because it was part of the outcome definition.

In the exploratory sensitivity analysis, among recipients recorded as receiving CMV at T0, 39 of 40 had complete exposure and outcome data. The coefficient estimates were negative in both the unadjusted model (β = −0.837, 95% CI −1.458 to −0.216, *P* = 0.0096) and the minimally adjusted model (β = −1.108, 95% CI −1.831 to −0.384, *P* = 0.0038), consistent in direction with the primary analysis (Supplementary Table S6).

### Stratum-specific heterogeneity analyses

In unadjusted stratum-specific analyses for the primary pressure-burden endpoint, the association between T0 dPBW-normalized tidal volume and T0 peak pressure difference was β = −0.503 (95% CI −0.961 to −0.046; *P* = 0.0319; *n* = 45) among single-lung transplant recipients and β = −0.595 (95% CI −1.270 to 0.079; *P* = 0.0819; *n* = 40) among bilateral lung transplant recipients. The unadjusted interaction by transplant type was not significant (interaction β = −0.092, 95% CI −0.875 to 0.691; *P* = 0.8160).

In recipients without postoperative ECMO within 24 h, the corresponding estimate was β = −0.501 (95% CI −1.122 to 0.120; *P* = 0.1110; *n* = 47), whereas in recipients with postoperative ECMO within 24 h, it was β = −0.569 (95% CI −1.161 to 0.023; *P* = 0.0589; *n* = 38). The unadjusted interaction by postoperative ECMO status was not significant (interaction β = −0.068, 95% CI −0.914 to 0.778; *P* = 0.8732).

### Dynamic compliance and gas-exchange findings

In the time-matched T0 dynamic compliance analysis, T0 dPBW-normalized tidal volume was associated with higher time-matched T0 dynamic compliance. This association was present in the unadjusted model (β = 3.803, 95% CI 2.827–4.778, *P* < 0.001), minimally adjusted model (β = 3.316, 95% CI 2.229–4.404, *P* < 0.001), and enhanced adjusted model (β = 3.066, 95% CI 1.702–4.429, *P* < 0.001; Table 3; Supplementary Figure S1A).

**Table 3:** Secondary associations between donor-size-normalized ventilation exposure metrics and T0 dynamic compliance.

Exposure and model	β	95% CI	*P*-value	*n*
T0 dPBW-normalized tidal volume				
Unadjusted	3.803	2.827–4.778	<0.0001	87
Minimally adjusted	3.316	2.229–4.404	<0.0001	87
Enhanced adjusted	3.066	1.702–4.429	<0.0001	87
dPBW/rPBW ratio				
Unadjusted	−16.599	−27.725 to −5.474	0.0039	87
Minimally adjusted	−14.888	−28.502 to −1.274	0.0325	87
Enhanced adjusted	−14.433	−29.485 to 0.619	0.0599	87
Mean 0–24 h dPBW-normalized tidal volume				
Unadjusted	4.154	3.173–5.135	<0.0001	87
Minimally adjusted	3.667	2.567–4.768	<0.0001	87
Enhanced adjusted	3.311	1.935–4.688	<0.0001	87

dPBW, donor-predicted body weight; rPBW, recipient-predicted body weight; CI, confidence interval; FiO_2_, fraction of inspired oxygen. Tidal-volume exposures are expressed in mL/kg dPBW. For tidal-volume exposure rows, β is expressed as the change in T0 dynamic compliance (mL/cmH_2_O) per 1 mL/kg dPBW higher tidal-volume exposure; for dPBW/rPBW ratio rows, β is expressed as the change in T0 dynamic compliance (mL/cmH_2_O) per 1.0-unit higher dPBW/rPBW ratio. Dynamic compliance includes tidal volume in its calculation and may therefore be mathematically coupled to dPBW-normalized tidal volume. The enhanced adjusted model included recipient age, recipient sex, body mass index, transplant type, preoperative primary diagnosis group, preoperative invasive mechanical ventilation, preoperative extracorporeal membrane oxygenation, postoperative extracorporeal membrane oxygenation within 24 h, ischemic time, intraoperative transfusion, intraoperative cardiopulmonary bypass or extracorporeal membrane oxygenation support, T0 positive end-expiratory pressure, T0 FiO_2_, and donor age.

When the dPBW/rPBW ratio was evaluated as a donor–recipient size-matching metric, a higher ratio was associated with lower T0 dynamic compliance in the unadjusted model (β = −16.599, 95% CI −27.725 to −5.474, *P* = 0.0039) and minimally adjusted model (β = −14.888, 95% CI −28.502 to −1.274, *P* = 0.0325). This association was attenuated after enhanced adjustment and was no longer significant (β = −14.433, 95% CI −29.485 to 0.619, *P* = 0.0599; Table 3).

In the sensitivity analysis using mean dPBW-normalized tidal volume during the first 24 postoperative h, mean 0–24 h dPBW-normalized tidal volume was associated with higher T0 dynamic compliance after enhanced adjustment (β = 3.311, 95% CI 1.935–4.688, *P* < 0.001; Table 3).

In the T24 time-matched validation analysis, T24 dPBW-normalized tidal volume was associated with higher T24 dynamic compliance after enhanced adjustment (β = 2.292, 95% CI 0.926–3.659, *P* = 0.0014; Supplementary Table S2). By contrast, T0 dPBW-normalized tidal volume was not associated with T0 PaO_2_/FiO_2_ ratio after enhanced adjustment (β = 1.379, 95% CI −14.979 to 17.737, *P* = 0.8665), and T24 dPBW-normalized tidal volume was not associated with T24 PaO_2_/FiO_2_ ratio after enhanced adjustment (β = 5.883, 95% CI −10.330 to 22.097, *P* = 0.4704).

Additional enhanced adjusted gas-exchange analyses showed no significant associations between time-matched dPBW-normalized tidal volume and FiO_2_ or PaO_2_ at either T0 or T24. T0 dPBW-normalized tidal volume was not associated with T0 PaCO_2_ after enhanced adjustment (β = −0.737, 95% CI −2.340 to 0.866, *P* = 0.3607). T24 dPBW-normalized tidal volume was associated with lower T24 PaCO_2_ (β = −1.635, 95% CI −2.551 to −0.719, *P* < 0.001; Supplementary Table S2).

### Secondary clinical outcomes

For severe PGD at 48–72 h, T0 dPBW-normalized tidal volume was associated with lower odds in the unadjusted model (OR = 0.782, 95% CI 0.604–0.989, *P* = 0.0484). This association was attenuated after adjustment and was not significant in the enhanced adjusted model (OR = 0.926, 95% CI 0.657–1.285, *P* = 0.6470; Supplementary Table S3). A similar attenuation was observed when mean 0–24 h dPBW-normalized tidal volume was used as the exposure. The enhanced adjusted association was not significant (OR = 0.892, 95% CI 0.620–1.255, *P* = 0.5171). The dPBW/rPBW ratio showed wide confidence intervals and was not significantly associated with severe PGD after enhanced adjustment (OR = 9.599, 95% CI 0.309–404.712, *P* = 0.2109).

For liberation from invasive mechanical ventilation within 28 days, T0 dPBW-normalized tidal volume was associated with higher odds in the unadjusted model (OR = 1.387, 95% CI 1.082–1.827, *P* = 0.0135). This association was not significant after enhanced adjustment (OR = 1.159, 95% CI 0.780–1.721, *P* = 0.4552). The corresponding enhanced adjusted associations were also not significant for the dPBW/rPBW ratio (OR = 3.301, 95% CI 0.087–176.843, *P* = 0.5315) or mean 0–24 h dPBW-normalized tidal volume (OR = 1.278, 95% CI 0.851–1.973, *P* = 0.2423; Supplementary Table S3).

For 30-day mortality, T0 dPBW-normalized tidal volume was associated with lower odds in the unadjusted and minimally adjusted models. This association was no longer significant after enhanced adjustment (OR = 0.803, 95% CI 0.488–1.278, *P* = 0.3628). Enhanced adjusted models also showed no significant associations for the dPBW/rPBW ratio (OR = 0.099, 95% CI 0.001–7.734, *P* = 0.2986) or mean 0–24 h dPBW-normalized tidal volume (OR = 0.743, 95% CI 0.419–1.230, *P* = 0.2693; Supplementary Table S3). The enhanced adjusted T0 dPBW-normalized tidal-volume estimates for the secondary clinical outcomes are summarized in Supplementary Figure S1B.

### Restriction and effect-modification analyses

Restriction analyses of the dynamic compliance association are summarized in Supplementary Table S4 and Supplementary Figure S2A. In the full cohort, the enhanced adjusted association between T0 dPBW-normalized tidal volume and T0 dynamic compliance was β = 3.066 (95% CI 1.702–4.429, *P* < 0.001). After excluding recipients who received postoperative ECMO within 24 h, the estimate was β = 2.141 (95% CI −0.275 to 4.557, *P* = 0.0805). After excluding recipients who received intraoperative cardiopulmonary bypass or ECMO support, the estimate was β = 1.897 (95% CI −0.950 to 4.743, *P* = 0.1829). When both perioperative extracorporeal support groups were excluded simultaneously, the estimate was β = 1.897 (95% CI −0.950 to 4.743, *P* = 0.1829).

In the ECMO interaction analysis for dynamic compliance, the interaction term for postoperative ECMO within 24 h was not significant (interaction β = 0.332, 95% CI −2.278 to 2.943, *P* = 0.8001). Simple-slope estimates were positive in both ECMO strata (Supplementary Table S4; Supplementary Figure S2B).

In donor–recipient size-matching analyses, seven recipients (8.0%) had marked donor undersizing (dPBW/rPBW < 0.8), 66 (75.9%) had approximate size matching (dPBW/rPBW 0.8–1.2), and 14 (16.1%) had marked donor oversizing (dPBW/rPBW > 1.2). The interaction between T0 dPBW-normalized tidal volume and the continuous dPBW/rPBW ratio was not significant in the unadjusted model (interaction β = 0.107, 95% CI −3.577 to 3.791, *P* = 0.9540) or the minimally adjusted model (interaction β = 0.333, 95% CI −3.536 to 4.203, *P* = 0.8643) (Supplementary Table S5; Supplementary Figures S2C, D).

## Discussion

In this retrospective single-center cohort study of lung transplant recipients, higher T0 dPBW-normalized tidal volume under clinician-selected ventilation was associated with lower T0 peak pressure difference after enhanced adjustment. Dynamic compliance analyses showed directionally consistent associations with higher time-matched dynamic compliance. Donor-size-normalized ventilation metrics did not show significant associations with severe PGD at 48–72 h, liberation from invasive mechanical ventilation within 28 days, or 30-day mortality after enhanced adjustment. Together, these findings describe an exploratory proximal observational association under clinician-selected ventilation and indicate that dPBW can be examined as a descriptive donor-size-normalized exposure metric for lung-transplant research, rather than as a basis for causal or clinical inference.

The main contribution of this study is the use of a donor-size-normalized denominator to describe early postoperative ventilation exposure. In conventional protective ventilation, tidal volume is usually indexed to predicted body weight derived from the patient being ventilated (17–19). Lung transplantation creates a distinct physiologic setting because the ventilated parenchyma is the donor lung rather than the recipient's native lung. When donor and recipient body sizes differ, the same absolute tidal volume may represent different relative mechanical exposure depending on whether it is indexed to rPBW or dPBW (23, 24, 28). In this context, dPBW-normalized tidal volume provides a donor-size-normalized measure of delivered ventilation relative to estimated graft size, separating the exposure metric from donor size *per se* and from any prescriptive ventilator setting.

Using T0 peak pressure difference as the primary outcome reduced direct structural dependence on tidal volume compared with dynamic compliance. The primary pressure-burden endpoint did not directly include tidal volume in its formula. However, because exposure and outcome were measured contemporaneously under clinician-selected ventilation, the inverse association should be interpreted descriptively and should not be taken to indicate that higher donor-size-normalized tidal volume physiologically reduces early pressure burden.

Peak pressure difference is a bedside pressure-burden metric based on peak airway pressure rather than plateau pressure. It is commonly available in retrospective records and can describe early pressure burden under usual-care ventilation, but it should not be equated with standard driving pressure or respiratory-system elastic load.

The observed inverse association should be interpreted in the context of clinician-selected T0 ventilation. At ICU admission, delivered or tolerated tidal volume, selected ventilatory mode, PEEP, ECMO strategy, and measured pressure burden may all have been shaped by the clinician's contemporaneous bedside assessment of graft quality, recruitability, pressure tolerance, and overall instability (21, 22, 41). This shared decision context may introduce reverse causation, residual confounding, and confounding by indication, which may partly explain why higher dPBW-normalized tidal volume was observed together with lower peak pressure-derived burden.

Dynamic compliance analyses provided supportive but structurally coupled information. Because dynamic compliance is calculated from tidal volume and pressure difference, concordant directionality is expected to align partly with the pressure-burden result. These analyses do not provide an independent test of elastic load or causality.

Clinical outcome analyses showed a pattern distinct from the proximal physiologic endpoints. Severe PGD, 28-day liberation from invasive mechanical ventilation, and 30-day mortality are distal endpoints shaped by competing risks and multiple perioperative and postoperative pathways. This high-acuity cohort included substantial preoperative ECMO, postoperative ECMO within 24 h, intraoperative CPB or ECMO support, preoperative invasive mechanical ventilation, and intraoperative transfusion, and severe PGD occurred in 53 of 87 recipients (60.9%). Reperfusion injury and PGD-related inflammatory or endothelial pathways can influence early graft function and gas exchange (5, 10, 11, 31). Perioperative extracorporeal support and postoperative recovery factors may further obscure the marginal contribution of any single ventilation-exposure metric to distal outcomes (32–35). The present results are therefore hypothesis-generating rather than clinically actionable.

Gas-exchange analyses were less consistent. Time-matched dPBW-normalized tidal volume was not associated with PaO_2_/FiO_2_ ratio, FiO_2_, or PaO_2_ at either T0 or T24 after enhanced adjustment. The main additional gas-exchange finding was the association between T24 dPBW-normalized tidal volume and lower T24 PaCO_2_. This finding may reflect differences in ventilation efficiency, carbon dioxide clearance, and clinician-selected ventilatory settings and should be interpreted cautiously. Oxygenation after lung transplantation is strongly influenced by edema, atelectasis, reperfusion injury, shunt physiology, and pulmonary vascular tone (10–12). In recipients receiving ECMO, extracorporeal support and clinician-selected ventilatory management may further influence measured gas-exchange variables (22, 41).

Donor–recipient size matching remains conceptually important in this study, but the absence of a statistically significant dPBW/rPBW interaction should not be interpreted as evidence against the conceptual rationale for donor-size-normalized ventilation (23, 24, 28). Interaction and main-effect questions are distinct. In our data, the donor–recipient size relationship may have influenced baseline postoperative respiratory-system behavior, including the directionally lower T0 dynamic compliance observed with higher dPBW/rPBW before enhanced adjustment, without detectably changing the slope relating T0 dPBW-normalized tidal volume to dynamic compliance across the observed size range. This distinction is particularly relevant because most recipients were approximately size-matched, whereas the markedly undersized and oversized strata were small, limiting precision for detecting non-linear or threshold heterogeneity.

Moreover, in this analysis, dPBW/rPBW should be regarded as a structural approximation rather than a direct measurement of the functional implanted lung volume available for ventilation after transplantation. The latent quantity is shaped not only by donor size but also by transplant type, native-lung contribution, postoperative edema, atelectasis, recruitment, and recipient thoracic mechanics. Accordingly, the non-significant interaction provides limited evidence regarding effect modification in this cohort and does not exclude the conceptual rationale for donor-size-normalized ventilation. Prior lung-transplant studies have linked donor–recipient size relationships to survival, PGD, donor-size-based ventilation exposure, and disease-specific outcome differences, suggesting that any size effect may be endpoint-specific rather than uniformly expressed as early dynamic compliance heterogeneity (25–28).

The ECMO restriction and interaction analyses further characterized the dynamic compliance findings. After exclusion of recipients who received postoperative ECMO within 24 h or intraoperative cardiopulmonary bypass/ECMO support, the association between T0 dPBW-normalized tidal volume and T0 dynamic compliance remained directionally positive but was no longer significant. This attenuation may reflect reduced sample size, wider confidence intervals, and clinical heterogeneity rather than a definitive absence of association. Recipients requiring ECMO or cardiopulmonary bypass differ systematically from those who do not (32, 33, 45). Extracorporeal support may also influence gas-exchange targets, ventilator settings, airway pressures, and clinician decision-making (22, 41).

These findings are most appropriately interpreted as informing how postoperative ventilation exposure may be described in observational lung-transplant research. Reporting tidal volume relative to donor and recipient body size may help characterize exposure to the newly implanted graft, but the present data do not establish clinical utility or support changes in ventilatory management. Early peak airway pressure-derived burden may be a feasible retrospective descriptor when plateau pressure is unavailable, but its physiologic and clinical relevance requires prospective validation (20, 36–38).

This study has several limitations. First, the retrospective observational design leaves the analysis vulnerable to reverse causation and confounding by indication, and precludes causal inference or prediction model development (44). Exposure and outcome were measured at the same T0 bedside moment under clinician-selected ventilation, so both may have been jointly shaped by real-time assessment of graft quality, pressure tolerance, recruitability, ECMO strategy, and instability (21, 22, 41); this decision process cannot be fully eliminated by routine regression adjustment. These factors may partly explain the observed inverse association between dPBW-normalized tidal volume and peak pressure-derived burden (Supplementary Figure S3). Second, the single-center sample was modest, limiting model stability, endpoint precision, complete-case analysis, interaction analyses, and formal subgroup inference. Complete-case analysis may also have introduced selection bias if missingness was related to clinical severity or ventilation management (42). The high-acuity clinical context further limits inference about severe PGD, liberation from invasive mechanical ventilation, and mortality, as these distal endpoints are influenced by multiple perioperative and postoperative pathways. Third, measurement limitations constrain interpretation: the primary endpoint was peak-pressure-derived rather than plateau-pressure-based and may reflect airway resistance, inspiratory flow, ventilation mode, secretions, airway caliber, endotracheal tube diameter, and patient–ventilator interaction, as well as elastic load. Static compliance, plateau pressure, standard driving pressure, transpulmonary pressure, and mechanical power were not systematically available (20, 36–38). Fourth, dynamic compliance analyses were mathematically coupled to tidal volume and cannot independently establish a mechanical effect. Fifth, routine records lacked standardized end-of-surgery bronchoscopy findings, secretion or thrombus burden, anastomotic stenosis, endotracheal-tube-diameter data, and clinical rationale for PEEP selection. In addition, although no graft volume reduction or surgical trimming was identified in this cohort, donor-estimated size and donor–recipient size ratio remain imperfect surrogates for the functional implanted lung volume, which likely governs the actual ventilatory load after transplantation (23, 24, 28).

Ventilatory mode may also introduce heterogeneity when interpreting the observed association because airway pressure is the controlled variable in pressure-controlled modes, whereas tidal volume is the controlled variable in volume-controlled modes. Although the CMV-restricted sensitivity analysis was directionally consistent with the primary analysis, its exploratory nature, modest stratum size, and clinician-selected mode assignment preclude definitive mode-specific inference and leave residual heterogeneity or bias possible.

Future studies should prospectively evaluate donor-size-normalized ventilation metrics using standardized ventilation protocols and repeated measurements of respiratory mechanics. Such studies should compare dPBW- and rPBW-based tidal-volume metrics and incorporate respiratory-mechanics outcomes less dependent on ventilator setting choices, including plateau pressure, standard driving pressure, transpulmonary pressure where feasible, and mechanical power (20, 36, 38). Measures of graft injury, including graft imaging, fluid balance, and biomarkers of ischemia–reperfusion injury, would provide complementary biologic context (10, 11). Larger multicenter cohorts or protocolized prospective studies are needed to evaluate whether donor-size-normalized ventilation metrics have reproducible physiologic or clinical relevance.

In this retrospective single-center cohort, T0 dPBW-normalized tidal volume under clinician-selected ventilation was associated with lower T0 peak airway pressure-derived pressure burden, whereas major early clinical outcomes did not show significant associations after enhanced adjustment. These exploratory findings are hypothesis-generating and should not be interpreted as evidence that higher dPBW-normalized tidal volume reduces pressure burden or as support for changing protective ventilation targets. Prospective, protocolized studies with plateau pressure measurements are required to evaluate the reproducibility and clinical relevance of donor-size-normalized ventilation metrics.

## Data Availability

The datasets presented in this article are not readily available because the individual-level clinical datasets contain potentially identifiable recipient and donor information and therefore cannot be made publicly available. De-identified analytic data may be made available to qualified researchers upon scientifically justified request to the corresponding authors, subject to approval by Shanghai Pulmonary Hospital and any required ethics or data-governance review. Data sharing will be limited to variables necessary to reproduce the reported analyses and will require appropriate data-use safeguards. Requests to access the datasets should be directed to Junrong Ding, 1805032@tongji.edu.cn.
