# Supplementary material for: Donor-size-normalized tidal volume and early peak airway pressure-derived pressure burden after lung transplantation: an exploratory single-center cohort study
Source: Front Med (Lausanne). 2026 Jul 23;13:1873272. doi: 10.3389/fmed.2026.1873272 (PMC13443038; doi:10.3389/fmed.2026.1873272)
Supplement: Supplementary file 1 [file Data_Sheet_1.pdf]

Supplementary Material

Supplementary Figure S1. Secondary physiologic and clinical outcome analyses

(A) Enhanced adjusted dynamic-compliance models, including T0 dPBW-normalized tidal volume, mean 0–24 h dPBW-normalized tidal volume, and T24 dPBW-normalized tidal volume. Points denote regression coefficient estimates and horizontal lines denote 95% confidence intervals. (B) Enhanced adjusted associations between T0 dPBW-normalized tidal volume and secondary clinical outcomes. Points denote odds ratios and horizontal lines denote 95% confidence intervals. dPBW, donor-predicted body weight; IMV, invasive mechanical ventilation; PGD, primary graft dysfunction; VT, tidal volume.

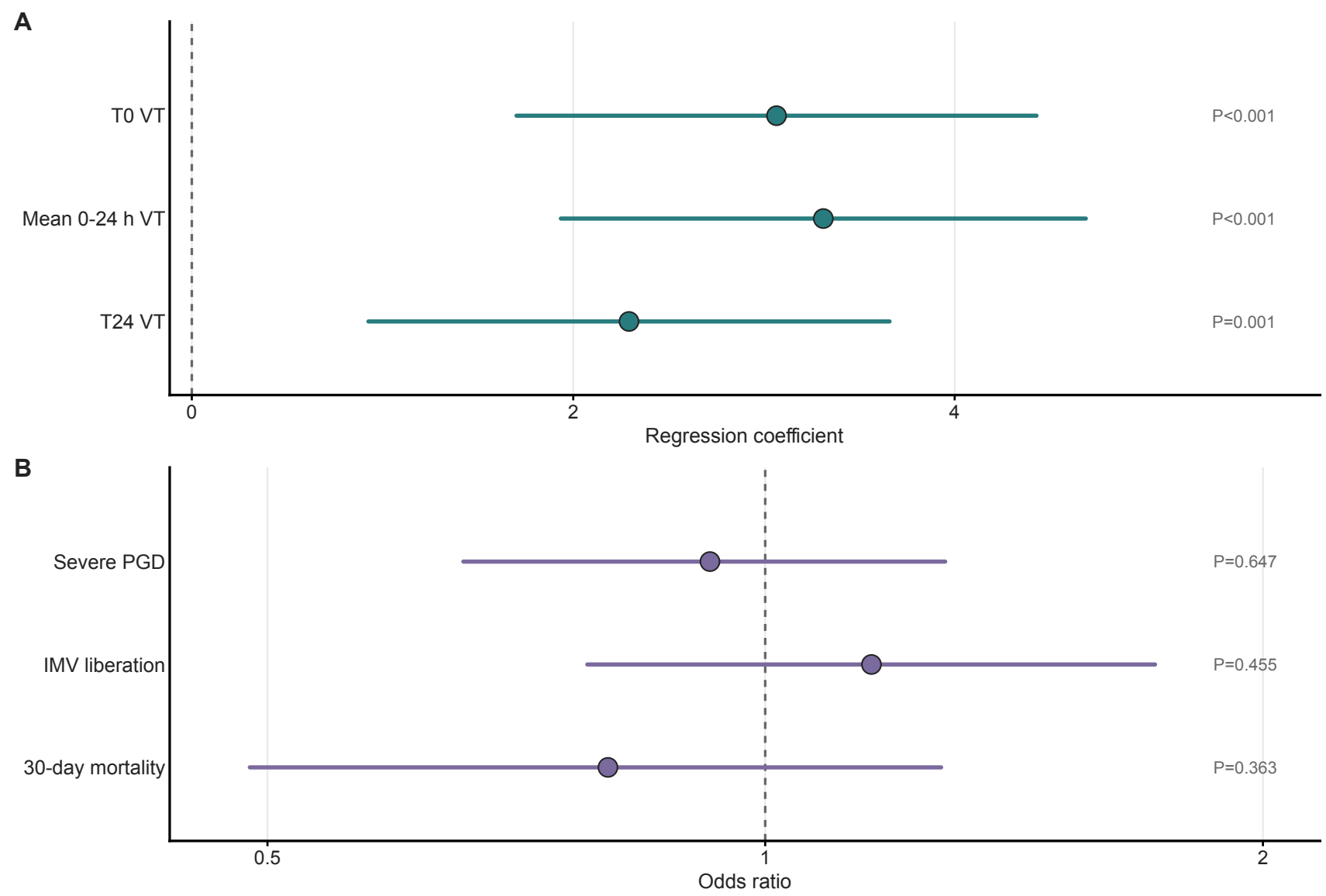

Supplementary Figure S2. Robustness and effect modification

(A) Restriction analyses for the association between T0 dPBW-normalized tidal volume and T0 dynamic compliance after excluding recipients with perioperative extracorporeal support. (B) Simple-slope estimates by postoperative ECMO status and the corresponding interaction test. (C) Distribution of donor–recipient size-matching categories. (D) Exploratory size-matching-stratified slopes. Points denote regression coefficient estimates and horizontal lines denote 95% confidence intervals. CPB, cardiopulmonary bypass; dPBW, donor-predicted body weight; ECMO, extracorporeal membrane oxygenation; rPBW, recipient-predicted body weight; VT, tidal volume.

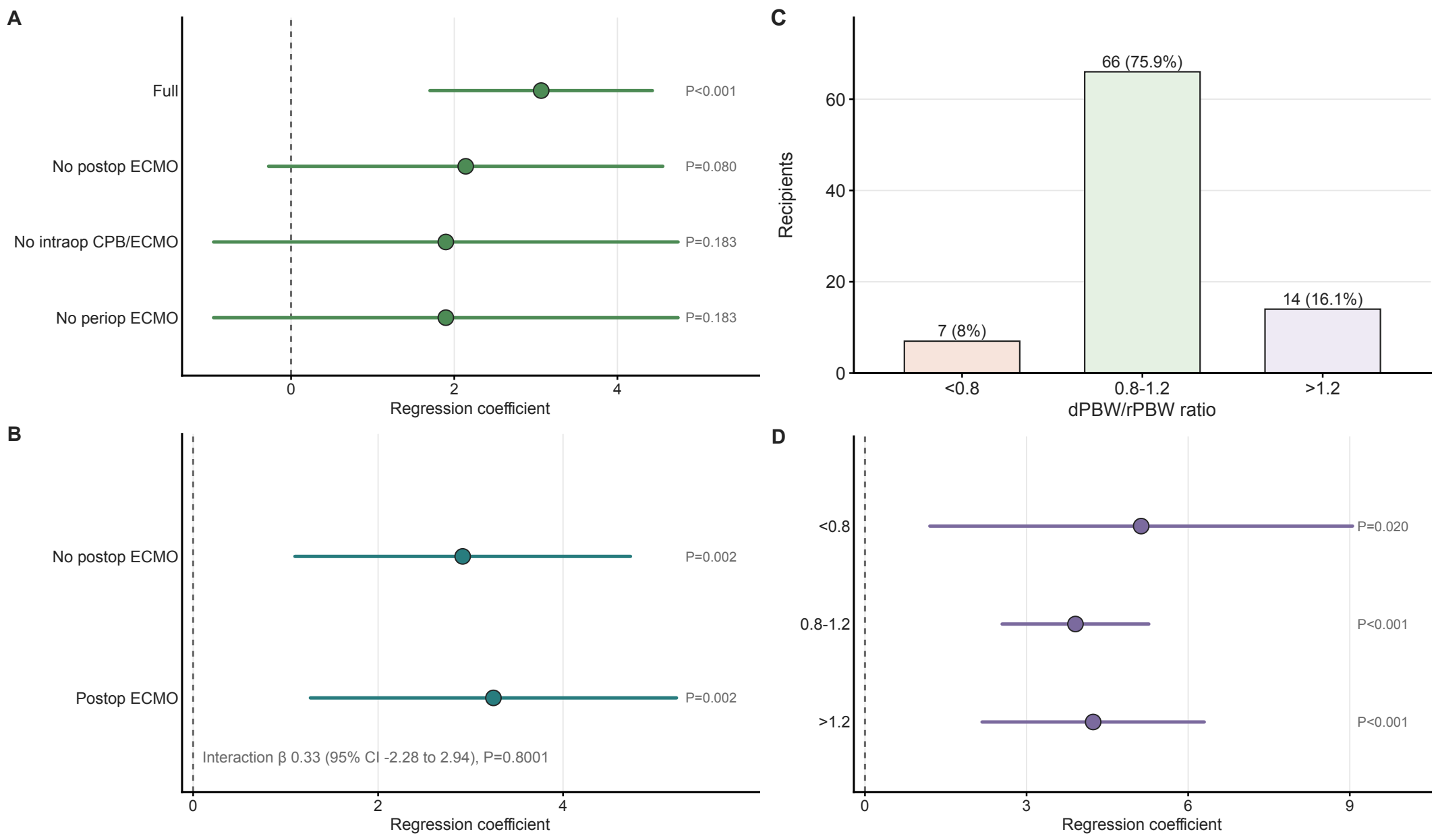

Supplementary Figure S3. Conceptual framework for dPBW-normalized tidal volume after lung transplantation

Conceptual framework showing donor body size, dPBW-normalized tidal volume, early peak airway pressure-derived burden, intermediate physiologic mechanisms, sources of heterogeneity and alternative explanations, and downstream clinical outcomes. Solid arrows show the primary observational exposure-to-outcome pathway. Dashed arrows show sources of heterogeneity and alternative explanations, including clinician-selected settings, ventilatory mode, PEEP, ECMO support, graft quality, airway factors, and reverse causation. This framework organizes plausible pathways and sources of bias; it is not a causal treatment model. CPB, cardiopulmonary bypass; dPBW, donor-predicted body weight; ECMO, extracorporeal membrane oxygenation; FiO_2, fraction of inspired oxygen; IMV, invasive mechanical ventilation; PEEP, positive end-expiratory pressure; PGD, primary graft dysfunction.

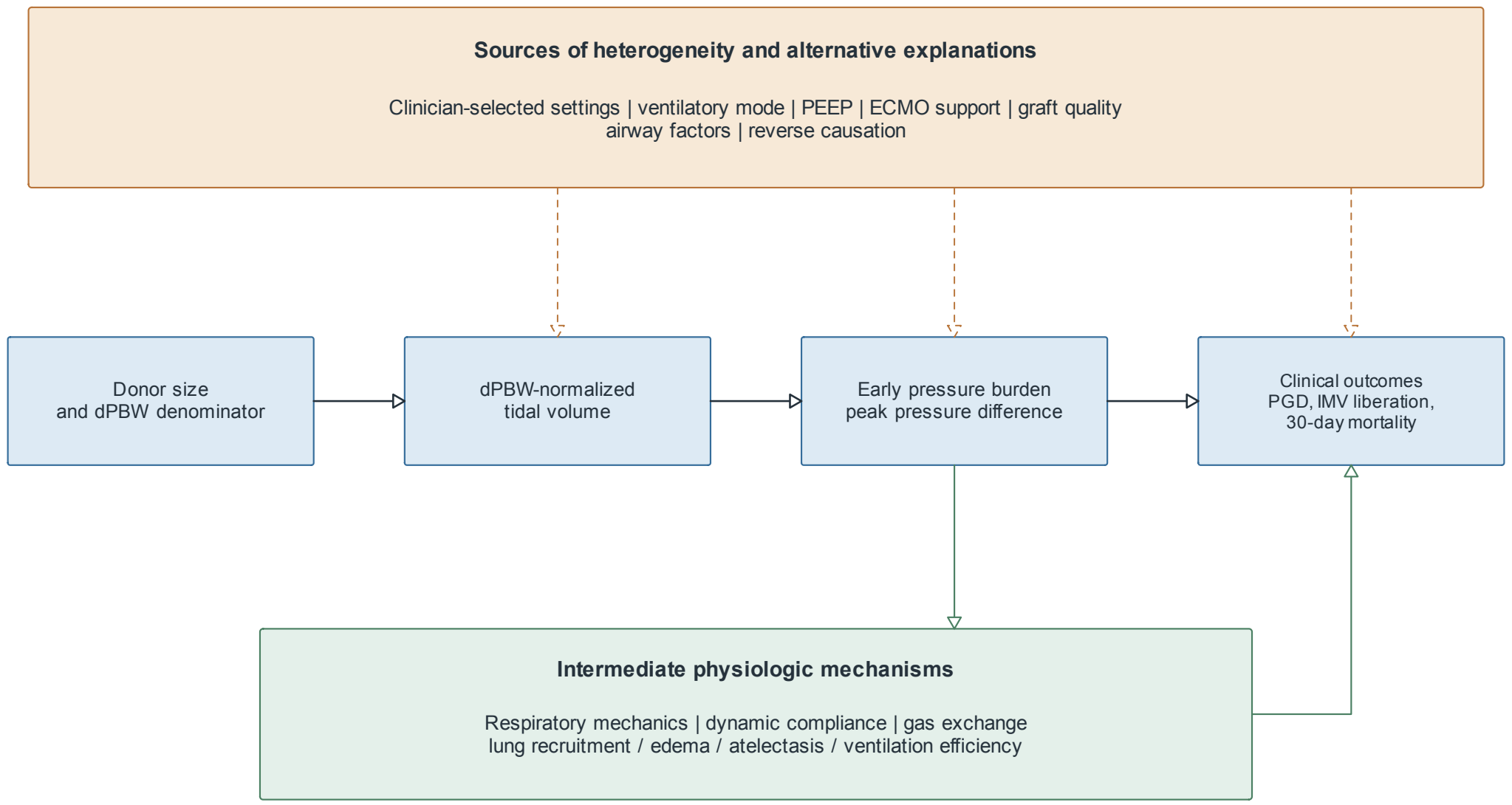

Supplementary Table S1 Missingness of key exposure, outcome, and covariate variables.

Variable or analysis requirement	Reference cohort, n	Missing, n	Available, n
Adult lung transplant recipients screened	92	–	92
Retransplantation exclusion status	92	0	92
Eligible recipients after exclusion of retransplantation	91	–	91
T0 dPBW-normalized tidal volume	91	4	87
T0 peak pressure difference	91	6	85
Covariates required for the primary enhanced model	91	6	85
Primary complete-case analytic cohort	91	6	85
T0 dynamic compliance for supportive analyses	91	4	87

dPBW, donor-predicted body weight. T0 denotes intensive care unit admission after transplantation. Six eligible recipients were excluded from the primary complete-case analysis because of missing T0 data required for the primary exposure, primary outcome, or model covariates. T0 dynamic compliance was available in 87 recipients and was analyzed as a supportive physiologic outcome. Complete-case analyses may be vulnerable to selection bias if missingness is related to clinical severity or ventilation management.

Supplementary Table S2 Enhanced adjusted time-matched validation and gas-exchange analyses.

Outcome	Exposure	β	95% CI	P value
**Time-matched validation**				
T24 dynamic compliance	T24 dPBW-normalized tidal volume	2.292	0.926 to 3.659	0.0014
T0 PaO_2/FiO_2 ratio	T0 dPBW-normalized tidal volume	1.379	-14.979 to 17.737	0.8665
T24 PaO_2/FiO_2 ratio	T24 dPBW-normalized tidal volume	5.883	-10.330 to 22.097	0.4704
**Gas-exchange outcomes**				
T0 FiO_2	T0 dPBW-normalized tidal volume	-0.798	-2.607 to 1.011	0.3820
T24 FiO_2	T24 dPBW-normalized tidal volume	-0.071	-1.006 to 0.863	0.8792
T0 PaO_2	T0 dPBW-normalized tidal volume	0.650	-6.607 to 7.908	0.8582
T24 PaO_2	T24 dPBW-normalized tidal volume	1.257	-4.621 to 7.135	0.6702
T0 $PaCO_2$	T0 dPBW-normalized tidal volume	-0.737	-2.340 to 0.866	0.3607
T24 $PaCO_2$	T24 dPBW-normalized tidal volume	-1.635	-2.551 to -0.719	0.0007

dPBW, donor-predicted body weight; CI, confidence interval; FiO_2, fraction of inspired oxygen; PaO_2, arterial oxygen tension; $PaCO_2$, arterial carbon dioxide tension. All estimates are from enhanced adjusted linear regression models. T0 denotes intensive care unit admission after transplantation; T24 denotes 24 hours after transplantation. The validation analyses evaluated time-matched associations between dPBW-normalized tidal volume and respiratory-mechanics or oxygenation endpoints.

Supplementary Table S3 Enhanced adjusted associations between donor-size-normalized ventilation metrics and secondary clinical outcomes.

Exposure metric	Outcome	OR	95% CI	P value	n
**T0 dPBW-normalized tidal volume**					
	Severe PGD at 48–72 h	0.93	0.66 to 1.28	0.6470	87
	Liberation from IMV within 28 days	1.16	0.78 to 1.72	0.4552	87
	30-day mortality	0.80	0.49 to 1.28	0.3628	87
**dPBW/rPBW ratio**					
	Severe PGD at 48–72 h	9.60	0.31 to 404.71	0.2109	87
	Liberation from IMV within 28 days	3.30	0.09 to 176.84	0.5315	87
	30-day mortality	0.10	0.00 to 7.73	0.2986	87
**Mean 0–24 h dPBW-normalized tidal volume**					
	Severe PGD at 48–72 h	0.89	0.62 to 1.25	0.5171	87
	Liberation from IMV within 28 days	1.28	0.85 to 1.97	0.2423	87
	30-day mortality	0.74	0.42 to 1.23	0.2693	87

dPBW, donor-predicted body weight; rPBW, recipient-predicted body weight; PGD, primary graft dysfunction; IMV, invasive mechanical ventilation; OR, odds ratio; CI, confidence interval. All estimates are from enhanced adjusted logistic regression models. Clinical outcomes were defined as severe PGD at 48–72 h, liberation from invasive mechanical ventilation within 28 days, and 30-day mortality. The wide confidence intervals for dPBW/rPBW ratio models indicate limited precision and should be interpreted cautiously.

Supplementary Table S4 Restriction and exploratory ECMO interaction analyses for the association between T0 dPBW-normalized tidal volume and T0 dynamic compliance.

Analysis cohort or contrast	β (95% CI)	P value	n
**Restriction analyses**			
Full cohort	3.066 (1.702 to 4.429)	<0.0001	87
Excluding postoperative ECMO within 24 h	2.141 (-0.275 to 4.557)	0.0805	47
Excluding intraoperative CPB/ECMO support	1.897 (-0.950 to 4.743)	0.1829	43
Excluding both intraoperative and postoperative ECMO support	1.897 (-0.950 to 4.743)	0.1829	42
**Exploratory ECMO interaction analysis: simple slopes**			
No postoperative ECMO within 24 h	2.915 (1.101 to 4.729)	0.0021	47
Postoperative ECMO within 24 h	3.247 (1.268 to 5.227)	0.0017	40
**Interaction term**			
T0 dPBW-normalized tidal volume × postoperative ECMO within 24 h	0.332 (-2.278 to 2.943)	0.8001	87

dPBW, donor-predicted body weight; ECMO, extracorporeal membrane oxygenation; CPB, cardiopulmonary bypass; CI, confidence interval. The restriction analyses refit the enhanced adjusted model after excluding recipients with perioperative extracorporeal support. Simple slopes are shown by postoperative ECMO status. The interaction term tested whether postoperative ECMO within 24 h modified the association between T0 dPBW-normalized tidal volume and T0 dynamic compliance.

Supplementary Table S5 Exploratory donor–recipient size-matching analysis by dPBW/rPBW category.

	dPBW/rPBW category		
	<0.8 Undersizing	0.8–1.2 Matched	>1.2 Oversizing
Recipients, n	7	66	14
Stratum-specific β (95% CI)	5.128 (1.204 to 9.051)	3.907 (2.546 to 5.269)	4.236 (2.174 to 6.298)
P value	0.0201	<0.0001	0.0008
**Continuous interaction: T0 dPBW-normalized tidal volume × dPBW/rPBW ratio**			
Unadjusted model	interaction β = 0.107 (95% CI -3.577 to 3.791), P = 0.9540		
Minimally adjusted model	interaction β = 0.333 (95% CI -3.536 to 4.203), P = 0.8643		

dPBW, donor-predicted body weight; rPBW, recipient-predicted body weight; CI, confidence interval. The stratum-specific estimates are exploratory because the marked undersizing and oversizing strata were small. The continuous interaction models evaluated whether the dPBW/rPBW ratio modified the association between T0 dPBW-normalized tidal volume and T0 dynamic compliance.

Supplementary Table S6 Additional exploratory checks for heterogeneity, ventilation mode, and PEEP.

Analysis	Subgroup or term	n	β	95% CI	P value
**A. CMV-restricted exploratory sensitivity analysis for T0 peak pressure difference**					
Unadjusted model	CMV at T0	39	-0.837	-1.458 to -0.216	0.0096
Minimally adjusted model	CMV at T0	39	-1.108	-1.831 to -0.384	0.0038
**B. Exploratory stratum-specific associations with T0 peak pressure difference**					
Single-lung transplantation stratum	Single-lung transplantation	45	-0.503	-0.961 to -0.046	0.0319
Bilateral-lung transplantation stratum	Bilateral-lung transplantation	40	-0.595	-1.270 to 0.079	0.0819
Transplant-type interaction	T0 dPBW-normalized tidal volume × bilateral transplantation	85	-0.092	-0.875 to 0.691	0.8160
No postoperative ECMO within 24 h stratum	No postoperative ECMO within 24 h	47	-0.501	-1.122 to 0.120	0.1110
Postoperative ECMO within 24 h stratum	Postoperative ECMO within 24 h	38	-0.569	-1.161 to 0.023	0.0589
Postoperative ECMO interaction	T0 dPBW-normalized tidal volume × postoperative ECMO within 24 h	85	-0.068	-0.914 to 0.778	0.8732

C. T0 ventilation mode distribution in the T0 base cohort

T0 ventilation mode	n	Percent
CMV	40	46.0
PCV	30	34.5
PSIMV	12	13.8
SIMV	5	5.7

D. T0 PEEP distribution in the T0 base cohort

Variable	Non-missing n	Missing n	Median	IQR	Range
T0 PEEP, cmH_2O	87	0	5.0	5.0 to 8.0	5.0 to 10.0

dPBW, donor-predicted body weight; ECMO, extracorporeal membrane oxygenation; PEEP, positive end-expiratory pressure; CMV, controlled mechanical ventilation; PCV, pressure-controlled ventilation; PSIMV, pressure synchronized intermittent mandatory ventilation; SIMV, synchronized intermittent mandatory ventilation; CI, confidence interval. T0 peak pressure difference was defined as T0 peak airway pressure minus T0 PEEP and was interpreted as a peak airway pressure-derived pressure-burden metric rather than standard driving pressure. β estimates represent the change in T0 peak pressure difference per 1 mL/kg higher T0 dPBW-normalized tidal volume. The minimally adjusted CMV-restricted model used the same minimally adjusted covariate set as the primary analysis. Enhanced adjustment was not fitted in the CMV-restricted analysis because of the limited stratum size. Stratum-specific and interaction analyses were unadjusted descriptive checks and are provided to describe heterogeneity across clinically relevant strata.
